# Expression differences and diagnostic efficacy of core plasma biomarkers in Alzheimer’s disease, cerebral small vessel disease and healthy adults

**DOI:** 10.3389/fneur.2026.1934127

**Published:** 2026-08-27

**Authors:** Wang Xiandong, Wu Junqi, Zhang Xuan, Ma Daichao, Zhao Xiaoyan, He Lihua, Zhang Hui

**Affiliations:** 1 The First School of Clinical Medicine, Shaanxi University of Chinese Medicine, Xianyang, China; 2 Department of Neurology, Affiliated Hospital of Shaanxi University of Chinese Medicine, Xianyang, China

**Keywords:** Alzheimer’s disease, cerebral small vessel disease, cognitive impairment, diagnostic efficacy, plasma biomarkers

## Abstract

**Objective:**

Alzheimer’s disease (AD) and cerebral small vessel disease (CSVD) are the two leading causes of cognitive impairment in the elderly, with overlapping clinical manifestations. This study aimed to explore the expression differences of plasma Aβ1-42, Aβ1-40, Aβ1-42/Aβ1-40, p-Tau181, p-Tau217, NfL and GFAP among patients with AD, CSVD and healthy populations, and to evaluate the diagnostic value of these biomarkers for AD as well as the differential diagnostic efficacy between AD and CSVD.

**Methods:**

A total of 120 participants were enrolled and divided into AD group, CSVD group and healthy control group, with 40 cases in each group. Plasma biomarkers were detected by chemiluminescence immunoassay, and cognitive function and neuroimaging examinations were completed simultaneously. The differences of biomarker levels among the three groups were compared. Spearman correlation analysis was used to analyze the correlation between each biomarker and MMSE score, and ROC curve was adopted to evaluate the diagnostic and differential diagnostic efficacy of single biomarker.

**Results:**

Plasma Aβ1-42 and Aβ1-42/Aβ1-40 ratio were significantly decreased, while p-Tau181 and p-Tau217 were markedly elevated in the AD group. The GFAP level in the CSVD group was specifically and significantly higher than that in the AD group and healthy control group. NfL was significantly increased in both disease groups. p-Tau217 exhibited optimal efficacy in distinguishing AD from healthy controls (AUC = 0.894) and differentiating AD from CSVD (AUC = 0.877). GFAP showed excellent diagnostic value in distinguishing CSVD from healthy controls (AUC = 0.881). All biomarkers were significantly correlated with MMSE scores.

**Conclusion:**

Among patients with isolated AD or isolated CSVD, plasma p-Tau217 is the optimal specific biomarker for the diagnosis of AD and differentiation between AD and cerebral small vessel disease. GFAP acts as a key indicator for identifying CSVD. These findings should be interpreted cautiously for patients with AD-CSVD co-pathology. Combined detection of multiple plasma biomarkers provides an important clinical basis for non-invasive early screening and etiological classification of cognitive impairment in patients with pathologically isolated cognitive disorders.

## 1 Introduction

With the acceleration of global population aging, cognitive impairment and dementia have become one of the most important public health challenges in the elderly population. AD is the most common type of dementia, clinically characterized by progressive memory decline, executive dysfunction, language disorder and decreased ability of daily living (1). In addition to neurodegenerative lesions, CSVD is also an important pathological basis for cognitive decline in the elderly, and serves as the main etiology of vascular cognitive impairment (VCI) (2). CSVD is caused by lesions of intracranial small arteries, venules, arterioles and capillaries, presenting a series of neuroimaging manifestations including white matter hyperintensities, lacunar infarction, cerebral microbleeds, enlarged perivascular spaces and brain atrophy (3). Epidemiological studies have shown that 30%–64% of CSVD patients suffer from varying degrees of cognitive impairment, suggesting its vital role in the occurrence and progression of dementia (4).

Cognitive impairment (CI) refers to the impairment of one or more cognitive domains including memory, attention, executive function, language and visuospatial ability (5). Both AD and CSVD can lead to cognitive decline, and they often coexist in the elderly with overlapping clinical and imaging features, which increases the complexity of etiological identification and differential diagnosis (6). Although neuropsychological assessment and neuroimaging examination remain the important basis of current clinical diagnosis, their sensitivity and specificity are limited in the early stage of the disease (7). Therefore, the development of simple, minimally invasive and repeatable peripheral blood biomarkers is of great clinical significance for improving the early identification and precise classification of cognitive impairment (8).

Aβ deposition and abnormal Tau hyperphosphorylation are the core pathological mechanisms of AD (9). Abnormal aggregation of Aβ forms extracellular amyloid plaques, while excessive phosphorylation of Tau protein promotes the formation of neurofibrillary tangles. Together, they drive synaptic dysfunction, neuronal injury and cognitive decline (10). In recent years, plasma Aβ1-42, Aβ1-40 and Aβ1-42/Aβ1-40 have been widely used to reflect cerebral amyloid pathological burden (11). Meanwhile, plasma p-Tau181 and p-Tau217 are well-studied peripheral phosphorylated tau biomarkers linked to AD pathophysiology, yet they do not directly mirror intraneuronal tau neurofibrillary tangle pathology; their elevations are predominantly driven by cerebral amyloid-β deposition (12). In the asymptomatic or mild cognitive impairment stages, p-Tau181/217 levels can elevate when Aβ-PET is positive but Tau-PET remains largely unremarkable, indicating that these markers more closely represent an Aβ-associated soluble tau phosphorylation response rather than a direct quantification of NFTs (13). Consequently, equating p-Tau181/217 simply as indicators “specifically reflecting isolated tau pathology” is inaccurate; they are better suited for identifying AD biological processes characterized by Aβ positivity and providing risk stratification information in the early stages of the disease (14).

In addition to AD-specific pathological biomarkers, neurofilament light chain (NfL) and glial fibrillary acidic protein (GFAP) also provide important supplementary information for the pathological classification of cognitive impairment (15). NfL reflects axonal injury and neurodegeneration, while GFAP indicates astrocyte activation and neuroinflammatory response (16) Existing studies have shown that these two biomarkers are not only associated with disease burden and cognitive decline in AD, but also sensitive to chronic ischemic brain injury and neurovascular unit damage caused by CSVD (17). Therefore, Aβ, p-Tau, NfL and GFAP correspond to different pathological dimensions including amyloid pathology, Tau pathology, axonal injury and glial activation, providing a new biological window for analyzing the similarities and differences between AD and CSVD.

Nevertheless, despite the rapid progress in research on AD plasma biomarkers, the expression characteristics of the above core biomarkers in CSVD and their value in differentiating AD from CSVD lack systematic evaluation (18). Especially in real-world clinical settings, AD and CSVD often coexist, and it is difficult to clarify the dominant pathological process merely relying on symptomatology and routine imaging. Clarifying the differential expression patterns of different plasma biomarkers under AD, CSVD and healthy status, and evaluating their relationships with cognitive impairment and imaging burden are of great significance for establishing clinically operable biological stratification strategies for cognitive impairment (19).

Based on the above background, participants with AD, CSVD and healthy controls were enrolled in this study. Plasma levels of Aβ1-42, Aβ1-40, Aβ1-42/Aβ1-40, p-Tau181, p-Tau217, NfL and GFAP were systematically detected. Combined with MMSE score and imaging indicators including MTA score, Fazekas score and lacunar infarction, comprehensive analysis was performed. This study aimed to clarify the differential expression characteristics of the above core plasma biomarkers among the three groups, explore their relationships with cognitive impairment and CSVD imaging burden, and evaluate their diagnostic efficacy for AD as well as the differential diagnostic value between AD and CSVD via ROC curve and combined model. We hypothesized that AD patients would present more significant Aβ metabolic imbalance and abnormal Tau phosphorylation, while CSVD patients tend to have elevated biomarkers related to axonal injury and glial activation. Compared with single indicator, multi-biomarker combined strategy is expected to improve the accuracy and clinical applicability of etiological classification for cognitive impairment.

## 2 Subjects and methods

### 2.1 Study participants

Participants were continuously enrolled from inpatient and outpatient departments of the First Encephalopathy Department, Affiliated Hospital of Shaanxi University of Chinese Medicine between June 2025 and April 2026. All subjects were divided into Alzheimer’s disease group (AD group), cerebral small vessel disease group (CSVD group) and healthy control group (HC group), with 40 cases in each group.

*AD inclusion criteria*: ① In accordance with the relevant diagnostic criteria for Alzheimer’s disease (20); ② Aged 40–85 years; ③ Completed cognitive function assessment, cranial MRI and plasma biomarker detection; ④ Brain white matter hyperintensities assessed by the Fazekas scale were scored 0–1 (none/mild lesions); ⑤ Subjects or their family members provided informed consent and voluntarily participated in the study.

*AD exclusion criteria*: ① Moderate to severe cerebral small vessel disease burden, including Fazekas scale score ≥2 (moderate confluent lesions to severe extensive confluent white matter hyperintensities), multiple cerebral microbleeds, and more than 3 lacunar infarctions; ② Combined with Parkinson’s disease, frontotemporal dementia, dementia with Lewy bodies, brain tumor, hydrocephalus and other neurological diseases; ③ Severe cardiac, hepatic, renal, pulmonary and coagulation dysfunction or malignant tumor; ④ History of mental illness, drug or alcohol dependence; ⑤ Incomplete clinical or imaging data.

*CSVD inclusion criteria*: ① Complied with STRIVE-2 international neuroimaging diagnostic criteria for CSVD (18); ② Cranial MRI presented at least one typical CSV imaging manifestation: white matter hyperintensities, lacunar infarction, cerebral microbleeds, enlarged perivascular spaces, superficial cortical siderosis; ③ Brain white matter hyperintensities assessed by the Fazekas scale were scored 2–3 (moderate confluent lesions to severe extensive confluent lesions); ④ Aged 40–85 years; ⑤ Completed cognitive function assessment, cranial MRI and plasma biomarker detection; ⑥ Subjects or their family members provided informed consent and voluntarily participated in the study.

*CSVD exclusion criteria*: ① Typical clinical phenotype of Alzheimer’s disease (core episodic memory impairment, chronic progressive cognitive decline); ② History of stroke, cerebral hemorrhage, craniocerebral trauma, intracranial infection and demyelinating diseases; ③ Severe cardiac, hepatic, renal and pulmonary insufficiency or malignant tumor; ④ History of mental illness, drug or alcohol dependence; ⑤ Incomplete clinical or imaging data.

*HC inclusion criteria*: ① Aged 40–85 years; ② Normal cognitive function without memory decline, behavioral abnormalities and neurological symptoms; ③ No obvious CSVD imaging manifestations and intracranial organic lesions on cranial MRI, with Fazekas scale score 0–1 (none/mild white matter hyperintensities); ④ Matched with case groups in region, age, gender and education level; ⑤ Signed written informed consent.

*HC exclusion criteria*: ① Any type of cognitive impairment, neurodegenerative disease, stroke or brain trauma history; ② Presence of moderate to severe white matter hyperintensities (Fazekas score ≥2) or other definite cerebral small vessel disease lesions; ③ Severe cardiac, hepatic, renal, pulmonary, metabolic diseases or malignant tumor; ④ History of mental illness, drug or alcohol dependence; ⑤ Unable to cooperate with blood collection, MRI or cognitive assessment.

This study was approved by the Ethics Committee of Affiliated Hospital of Shaanxi University of Chinese Medicine (Approval No. SZFYIEC-PJ-2025 [51]).

### 2.2 Research methods

#### 2.2.1 General data collection

General demographic and clinical data were collected, including age, gender, educational background, years of education, occupation, marital status and living conditions. Lifestyle data such as smoking, drinking, physical activity and sleep status were recorded, and medical histories such as hypertension, diabetes and dyslipidemia were also collected.

#### 2.2.2 Cognitive function assessment

The Mini-Mental State Examination (MMSE) was used to evaluate cognitive function in all subjects. The total score of MMSE was 30 points, including time orientation, place orientation, immediate memory, attention and calculation, delayed recall, language ability and visuospatial ability. MMSE score ≥24 indicated normal cognition, while score <24 suggested cognitive impairment. All scale assessments were completed by uniformly trained researchers.

#### 2.2.3 Neuroimaging assessment

Cranial MRI data were collected to record CSVD-related imaging manifestations including white matter hyperintensities, lacunar infarction and brain atrophy. The medial temporal lobe atrophy (MTA) scale was used to assess the degree of medial temporal lobe atrophy, and the Fazekas scale was adopted to evaluate the severity of white matter hyperintensities. The presence of lacunar infarction was recorded simultaneously. Imaging evaluation was independently performed by two senior radiologists blinded to clinical data, and consensus was reached through negotiation in case of inconsistency.

#### 2.2.4 Plasma biomarker detection

Plasma levels of Aβ1-42, Aβ1-40, p-Tau181, p-Tau217, GFAP and NfL were detected by automatic chemiluminescence immunoanalyzer (Shine i800, Serial No. A291040044182), and the Aβ1-42/Aβ1-40 ratio was calculated according to standard operating procedures.

(1) *Blood sample collection*: A total of 5 mL venous blood was collected with EDTA anticoagulant tube, and fasting was not required before collection. The tube was gently inverted and mixed several times to ensure sufficient anticoagulation.
(2) *Sample processing and storage*: Samples detected within 8 h were stored at room temperature or 2 °C–8 °C. Samples not detected within 8 h were centrifuged at 4,000 rpm/min (2,500 × *g*) for 10 min at room temperature of about 22 °C to separate upper plasma. Samples with obvious hemolysis or lipemia were discarded. The separated plasma was transferred to 1.5 mL centrifuge tube and stored at −20 °C or below. Only one freeze–thaw cycle was allowed for each sample. After thawing, the plasma was gently mixed and centrifuged at 10,000 rpm/min (11,190 × *g*) for 5 min before detection.
(3) *Instrument calibration and quality control*: Instrument calibration and internal quality control were completed before detection in accordance with the manufacturer’s instructions to ensure stable detection performance.
(4) *On-board detection*: Each sample required no less than 600 μL plasma without obvious suspended matter or precipitation, and the detection was strictly performed in accordance with operating specifications.
(5) *Process recording*: The whole process of blood collection, processing, storage, freeze–thaw and detection was recorded in detail to ensure detection quality and evaluate the potential influence of pre-analytical factors on results.

### 2.3 Statistical analysis

SPSS 27.0 software was used for statistical analysis. The Shapiro–Wilk test was used to test the normality of measurement data. Normally distributed data were expressed as mean ± standard deviation, and one-way ANOVA was used for multi-group comparison with Bonferroni *post hoc* test for pairwise comparisons (all pairwise *p* values adjusted by Bonferroni correction to control Type I error). Non-normally distributed data were presented as median [M(P25, P75)], and Kruskal–Wallis H test was adopted for multi-group comparison. Enumeration data were described as case number and percentage, and compared by χ^2^ test or Fisher’s exact test.

Spearman correlation analysis was used to evaluate the correlations of plasma Aβ1-42, Aβ1-40, Aβ1-42/Aβ1-40, p-Tau181, p-Tau217, GFAP, NfL with MMSE score, MTA score, Fazekas score and lacunar infarction; all correlation *p* values were adjusted via Bonferroni correction for multiple testing. Binary Logistic regression models incorporating age and gender as covariates were built for each single plasma biomarker to generate age- and gender-adjusted predicted probabilities, which were then used to plot ROC curves to evaluate the diagnostic efficacy of single biomarkers for AD, with focus on the differential diagnostic value between AD and CSVD. The area under the curve (AUC) was used to evaluate diagnostic efficiency, and pairwise comparisons of AUC across biomarkers were performed via DeLong test, with all DeLong-derived *p* values corrected using Bonferroni method. The optimal cutoff values for each plasma biomarker were determined by maximizing the Youden index (J = Sensitivity + Specificity − 1) using SPSS 27.0. The corresponding sensitivity, specificity, and 95% confidence intervals of AUC were reported; Bootstrap resampling (*n* = 1,000) was performed to validate the stability of cutoff values. Sensitivity analysis was performed to test the influence of potential outliers on intergroup comparison results of plasma biomarkers. All *p* values presented in tables, figures and main text refer to Bonferroni-corrected p values after multiple comparison adjustment. A corrected *p* value < 0.05 was considered statistically significant.3 Results.

### 2.4 Demographic and clinical characteristics of all participants

The demographic, clinical, biochemical and imaging characteristics among different diagnostic groups are shown in Table 1. A total of 120 subjects were enrolled, including 40 cases in healthy control (HC) group, CSVD group and AD group, respectively. There were no significant differences in age and BMI among the three groups (*p* = 0.293, *p* = 0.471). Gender composition and years of education showed statistically significant differences (both *p* = 0.002). No significant differences were observed in smoking, drinking, physical inactivity and insomnia among the three groups (all *p* > 0.05).

**Table 1:** Comparison of baseline demographic, clinical, biochemical and imaging characteristics among groups.

Variables	HC group (*n* = 40)	CSVD group (*n* = 40)	AD group (*n* = 40)	*p* value
Demographic characteristics				
Age (years), Median (Q1, Q3)	70.00 (67.00, 72.25)	74.00 (65.25, 78.25)	72.50 (63.00, 78.00)	0.293
Gender (Male/Female), *n*	10/30	25/15^a,b^	14/26^b^	0.002
Education years (years), M(Q1, Q3)	15.50 (12.00, 19.00)	12.00 (10.00, 14.25)^a^	12.00 (11.75, 15.00)^a^	0.002
BMI (kg/m^2^), Mean ± SD	22.85 ± 3.01	22.85 ± 3.38	22.07 ± 3.36	0.471
Lifestyle and sleep status, *n* (%)				
Smoking history (Smokers)	4 (10.0)	7 (17.5)	8 (20.0)	0.444
Alcohol drinking history (Drinkers)	5 (12.5)	6 (15.0)	3 (7.5)	0.568
Physical activity (Inactivity)	15 (37.5)	17 (42.5)	25 (62.5)	0.060
Sleep status (Insomnia)	15 (37.5)	20 (50.0)	15 (37.5)	0.424
Vascular risk factors, *n* (%)				
Hypertension (Yes)	8 (20.0)	20 (50.0)^a^	15 (37.5)	0.019
Diabetes mellitus (Yes)	6 (15.0)	11 (27.5)	5 (12.5)	0.178
Hyperlipidemia (Yes)	5 (12.5)	2 (5.0)	1 (2.5)	0.175
Coronary heart disease (Yes)	3 (7.5)	3 (7.7)	1 (2.5)	0.536
Arrhythmia (Yes)	6 (15.0)	7 (17.5)	5 (12.5)	0.822
Biochemical indicators, Median (Q1, Q3) / Mean ± SD				
Blood glucose (mmol/L)	5.54 (5.06, 5.82)	6.35 (5.70, 8.00)^a,b^	5.71 (5.38, 6.85)^a,b^	<0.001
TC (mmol/L)	4.65 ± 0.86	3.45 ± 0.79^a,b^	4.11 ± 1.35^a,b^	<0.001
LDL-C (mmol/L)	2.69 (2.36, 3.15)	1.92 (1.33, 2.26)^a,b^	2.17 (1.74, 2.77)^a,b^	<0.001
Hcy (umol/L)	14.00 (10.09, 16.27)	15.40 (14.00, 19.50)^a^	16.65 (11.40, 26.17)	0.027
Cognitive assessment, Median (Q1, Q3)				
MMSE score	27.00 (26.00, 28.25)	24.00 (22.00, 25.00)^a,b^	13.00 (11.00, 14.25)^a,b^	<0.001
Imaging characteristics, *n* (%)				
Carotid artery ultrasound				<0.001
0 (Normal)	24 (60.0)	7 (17.5)	15 (37.5)	
1 (Intima-media sclerosis/thickening)	10 (25.0)	0 (0.0)	1 (2.5)	
2 (Sclerosis combined with plaque formation)	6 (15.0)	29 (72.5)	24 (60.0)	
3 (Arterial stenosis)	0 (0.0)	4 (10.0)	0 (0.0)	
Fazekas scale (White matter hyperintensities)				<0.001
Score 0–1 (None/mild)	40 (100.0)	0 (0.0)	35 (87.5)	
Score 2 (Moderate, confluent lesions)	0 (0.0)	33 (82.5)	0 (0.0)	
Score 3 (Severe, extensive confluent lesions)	0 (0.0)	7 (17.5)	0 (0.0)	
Abnormal MTA score (≥2 points)*	0 (0.0)	4 (10.0)	40 (100.0)^a,b^	<0.001

Data are presented as mean (standard deviation) unless otherwise specified. Continuous variables were compared using one-way analysis of variance (ANOVA) and Kruskal–Wallis test. Categorical variables were analyzed with Pearson’s χ^2^ test and Fisher’s exact test. Analysis of covariance (ANCOVA) adjusted for age, gender and education level was applied for cognitive test scores. Raw scores are reported for MMSE. HC, healthy control group; CSVD, cerebral small vessel disease; AD, Alzheimer’s disease; TC, total cholesterol; LDL-C, low-density lipoprotein cholesterol; Hcy, homocysteine; BMI, body mass index; MMSE, Mini-Mental State Examination; MTA, medial temporal atrophy. a: *p* < 0.05 vs. HC group; b: *p* < 0.05 vs. AD group or CSVD group. *p < 0.05, **p < 0.01, ***p < 0.001, Bonferroni‑corrected *p*‑values.

In terms of vascular risk factors, the prevalence of hypertension in CSVD group was significantly higher than that in HC group (*p* = 0.019). No significant differences were found in the prevalence of diabetes, dyslipidemia, coronary heart disease and arrhythmia among three groups (all *p* > 0.05). Significant overall differences were detected in blood glucose, total cholesterol (TC), low-density lipoprotein cholesterol (LDL-C) and homocysteine (Hcy) levels. Blood glucose levels in CSVD and AD groups were significantly higher than those in HC group (*p* < 0.001). TC and LDL-C levels in CSVD group were significantly lower than those in HC and AD groups (all *p* < 0.001). In addition, Hcy levels in CSVD and AD groups were significantly higher than those in HC group (*p* = 0.027).

After adjustment for age, gender and education level by covariance analysis, MMSE scores were significantly different among three groups (*p* < 0.001). *Post-hoc* comparison showed that the median MMSE score in AD group (13.00) was significantly lower than that in CSVD group (24.00) and HC group (27.00). Significant overall differences were also observed in carotid ultrasound, Fazekas score and MTA score among three groups (all *p* < 0.001). Specifically, CSVD group presented the most severe cerebrovascular burden, with higher rates of carotid atherosclerosis complicated with plaque formation (72.5%) and moderate to severe white matter hyperintensities (Fazekas score ≥ 2 points, 100.0%) than other two groups. In terms of brain atrophy, the proportion of abnormal MTA score (≥2 points) in AD group reached 100.0%, which was significantly higher than 10.0% in CSVD group and 0.0% in HC group.

### 2.5 Plasma biomarker levels among different groups

**Figure 1:**
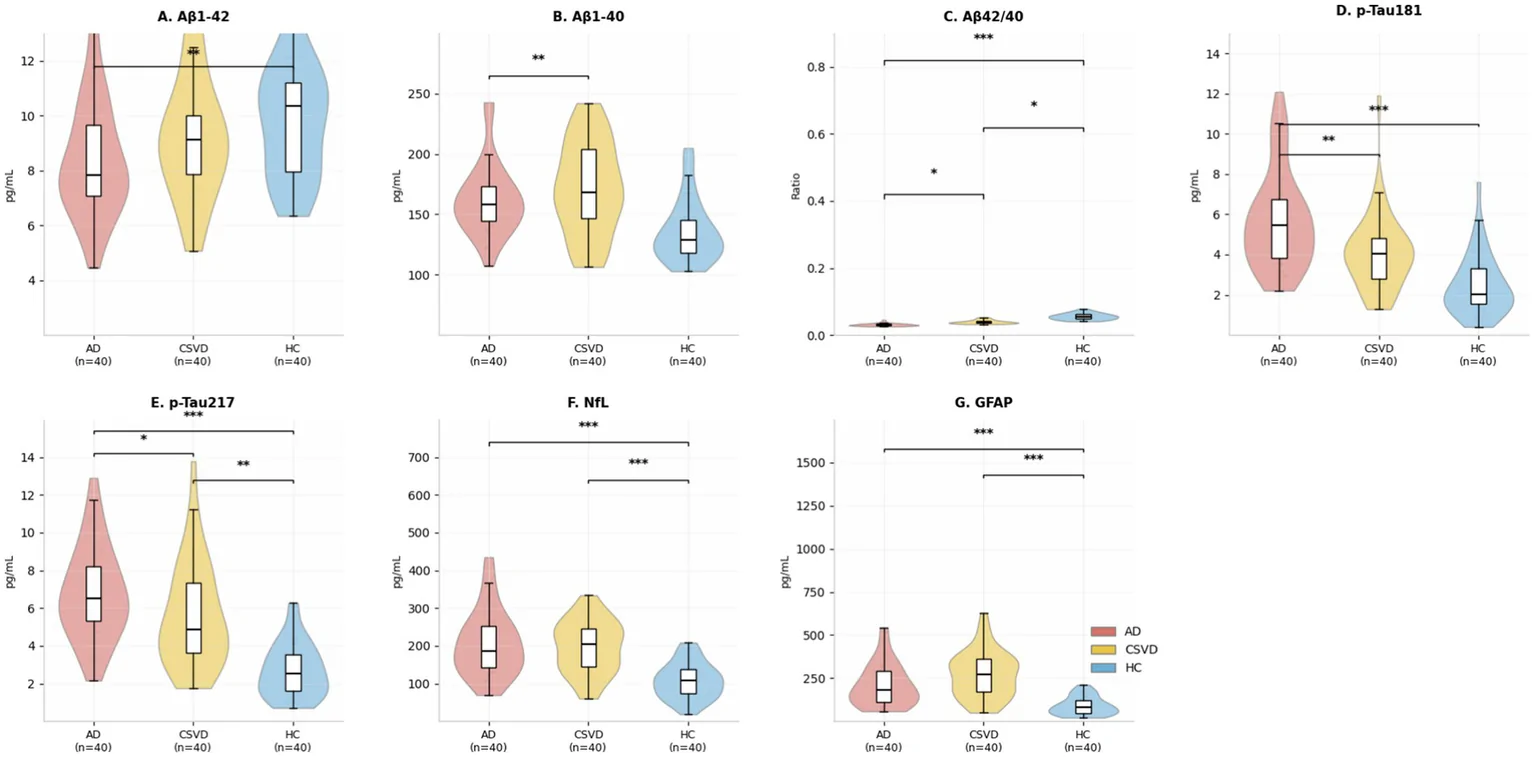
Plasma biomarker levels across AD, CSVD, and HC groups. **(A–G)** shows the levels/ratios of plasma A*β*1-42 **(A)**, Aβ1-40 **(B)**, Aβ1-42/Aβ1-40 ratio **(C)**, p-Tau181 **(D)**, p-Tau217 **(E)**, NfL **(F)**, and GFAP **(G)** in three groups of participants. Violin plots illustrate the kernel density distribution of the data. The translucent scattered dots inside represent individual raw data from each participant, and the thick black horizontal line in the middle indicates the mean of each group. Notably, one obvious outlier was observed in the healthy control (HC) group for the Aβ1-42/Aβ1-40 ratio, which partially affected the intergroup statistical differences among AD, CSVD, and HC groups. *p < 0.05, **p < 0.01, ***p < 0.001.

After adjusting for age and gender, the plasma levels of all biomarkers among the three groups are presented in Table 2 and Figure 1. One-way analysis of variance (ANOVA) showed that all detected biomarkers exhibited statistically significant overall differences across the three groups (all *p* < 0.05). Post-hoc pairwise comparisons revealed distinctly different plasma biomarker profiles between AD and CSVD. All plasma biomarker data conformed to normal distribution, hence all values are reported as mean ± standard deviation.

**Table 2:** Plasma biomarker levels across diagnostic groups.

Variables biomarkers (pg/mL)	HC group (*n* = 40) (Mean ± SD)	CSVD group (*n* = 40) (Mean ± SD)	AD group (*n* = 40) (Mean ± SD)	Corrected *p* value
Aβ1-42	7.06 ± 1.70	6.32 ± 1.71	5.67 ± 1.78^a^	0.0022**
Aβ1-40	115.51 ± 19.31	120.98 ± 33.44	135.19 ± 33.33^a^	0.0103*
Aβ1-42 / Aβ1-40	0.0757 ± 0.1052	0.0560 ± 0.0215	0.0442 ± 0.0182^a,b^	<0.001***
p-Tau181	1.652 ± 0.688	2.572 ± 1.146^a^	3.523 ± 2.439^a^	<0.001
p-Tau217	1.565 ± 1.906	2.107 ± 1.252	3.769 ± 2.700^a,b^	<0.001
NfL	68.389 ± 46.719	127.107 ± 96.088^a,b^	156.469 ± 83.913^a^	<0.001
GFAP	34.281 ± 34.860	180.876 ± 262.516^a,b^	79.412 ± 76.144^a^	<0.001

All continuous variables are expressed as mean ± standard deviation (Mean ± SD). Of note, GFAP exhibited skewed distribution with large dispersion in the CSVD group. One-way analysis of variance (ANOVA) was used for overall intergroup comparisons, and Bonferroni *post-hoc* test was applied for pairwise comparisons between groups. All *p* values were adjusted by Bonferroni correction for multiple comparisons. a: *p* < 0.05 vs. the healthy control group; b: *p* < 0.05 vs. the AD group or CSVD group. Aβ, amyloid-β; p-Tau, phosphorylated tau protein; NfL, neurofilament light chain; GFAP, glial fibrillary acidic protein. *p < 0.05, **p < 0.01, ***p < 0.001, Bonferroni‑corrected *p*‑values.

The AD group displayed typical amyloid and Tau pathological characteristics, with significantly reduced plasma Aβ1-42 levels and Aβ1-42/Aβ1-40 ratios. This ratio was markedly lower than that in the HC and CSVD groups (*p* < 0.05). Although p-Tau181 was elevated in both disease groups relative to healthy controls, p-Tau217 was highly and specifically overexpressed in the AD group, being significantly higher than in the HC and CSVD groups (*p* < 0.05).

Notably, biomarkers related to neurodegeneration and glial activation reflected the unique pathological features of CSVD. Plasma NfL levels in the CSVD group were significantly higher than those in the HC group, following a clear increasing gradient: HC < CSVD < AD (all *p* < 0.05). Most prominently, GFAP, a marker of astrocyte activation, was highly elevated in the CSVD group at 180.876 ± 262.516 pg./mL, which was significantly higher than both the HC and AD groups (*p* < 0.05). This marked elevation in GFAP indicates that intense astrocyte-mediated neuroinflammation serves as a key peripheral biomarker for distinguishing CSVD from AD.

Notably, GFAP exhibited markedly large standard deviation in the CSVD group (180.876 ± 262.516 pg./mL). This wide dispersion originates from heterogeneous astrocyte activation burden across CSVD patients: individuals with severe extensive white matter hyperintensities, multiple lacunar infarcts and recurrent cerebral microbleeds presented drastically elevated GFAP, while mild CSVD cases showed mild GFAP elevation, leading to broad data distribution as visualized in Figure 1G violin plot.

Notably, one high outlier of Aβ1-42/Aβ1-40 was identified in the healthy control group (Figure 1C). To verify the robustness of intergroup differences, we conducted a sensitivity analysis by removing this outlier. The results showed that the overall difference of Aβ1-42/Aβ1-40 ratio among the three groups was still statistically significant (*p* < 0.001). This outlier was derived from an elderly healthy participant with extremely high plasma Aβ1-42, relatively low Aβ1-40, and no cerebral amyloid deposition on cranial MRI.

### 2.6 Diagnostic and differential diagnostic efficacy of plasma biomarkers

**Figure 2:**
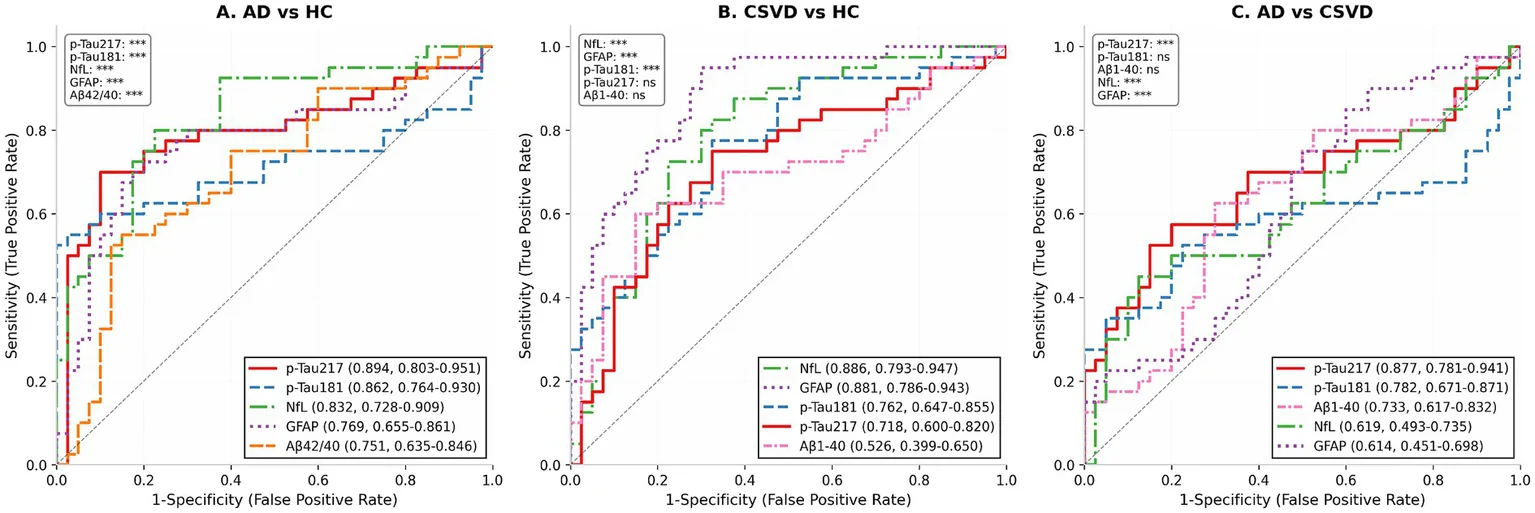
Receiver operating characteristic (ROC) curve analysis of plasma biomarkers across diagnostic groups. **(A)** Diagnostic performance for distinguishing Alzheimer’s disease (AD) from healthy controls (HC). All five biomarkers showed significantly elevated plasma levels in AD compared with HC (Bonferroni *post-hoc* test, all **p < 0.001). Plasma p-Tau217 exhibited the highest discriminative accuracy (*AUC* = 0.894, 95% *CI*: 0.803–0.951), followed by p-Tau181 (*AUC* = 0.862, 95% *CI*: 0.764–0.930). **(B)** Diagnostic performance for distinguishing cerebral small vessel disease (CSVD) from HC. NfL (*AUC* = 0.886, 95% *CI*: 0.793–0.947), GFAP (*AUC* = 0.881, 95% *CI*: 0.786–0.943), and p-Tau181 (*AUC* = 0.762, 95% *CI*: 0.647–0.855) showed significantly elevated plasma levels in CSVD compared with HC (Bonferroni post-hoc test, all **p < 0.001), whereas p-Tau217 and Aβ1-40 exhibited no significant intergroup differences. **(C)** Diagnostic performance for distinguishing AD from CSVD. p-Tau217 achieved the best differential efficacy (*AUC* = 0.877, 95% *CI*: 0.781–0.941), with significantly elevated levels in AD compared with CSVD (Bonferroni post-hoc test, **p < 0.001). NfL and GFAP also showed significant differences between AD and CSVD (both **p < 0.001), whereas p-Tau181 and A*β*1-40 exhibited no significant intergroup differences. The diagonal dashed line indicates the reference line for random classification (*AUC* = 0.5). Cutoff values were determined by maximizing the Youden index. Significance stars in each panel denote Bonferroni *post-hoc* test results for plasma level differences between the compared groups: *p < 0.05, **p < 0.01, ***p < 0.001; ns, not significant. Aβ, amyloid-β protein; p-Tau, phosphorylated tau protein; NfL, neurofilament light chain; GFAP, glial fibrillary acidic protein; AUC, area under the curve; CI, confidence interval.

After adjusting for age and gender, we further evaluated the diagnostic and differential diagnostic performance of each plasma biomarker using the receiver operating characteristic (ROC) curve and area under the curve (AUC) (Table 3; Figure 2). The results revealed distinct characteristic patterns across different clinical scenarios, which were consistent with the differential expression profiles observed in Table 2.

**Table 3:** Diagnostic efficacy of individual plasma biomarkers assessed by ROC curve analysis.

Diagnostic comparison setting	Plasma biomarker	AUC	95% CI	Cutoff (pg/mL)	Sensitivity (%)	Specificity (%)
Distinguishing Alzheimer’s disease from HC	p-Tau217	0.894	0.803–0.951	2.17	0.700	100.0
	p-Tau181	0.862	0.764–0.930	1.82	0.775	96.7
	NfL	0.832	0.728–0.909	83.21	0.800	77.5
	GFAP	0.769	0.655–0.861	49.53	67.5	85.0
	Aβ42/40	0.751	0.635–0.846	0.075	95.00	25.0
	Aβ1-40	0.699	0.579–0.803	130.2	60.0	85.00
	Aβ1-42	0.584	0.457–0.704	10.80	25.0	90.00
Distinguishing CSVD from HC	GFAP	0.881	0.786–0.943	36.39	95.0	70.0
	NfL	0.886	0.793–0.947	84.35	72.5	77.5
	p-Tau181	0.762	0.647–0.855	1.82	77.5	67.5
	p-Tau217	0.718	0.600–0.820	1.45	75.0	67.5
	Aβ1-40	0.526	0.399–0.650	130.8	32.5	85.0
	Aβ42/40	0.414	0.293–0.544	0.0718	22.5	92.5
	Aβ1-42	0.376	0.231–0.457	4.00	95.0	71.5
Distinguishing Alzheimer’s disease from CSVD	p-Tau217	0.877	0.781–0.941	3.07	52.5	85.0
	p-Tau181	0.782	0.671–0.871	3.24	52.5	77.5
	Aβ1-40	0.733	0.617–0.832	123.7	70.0	62.5
	NfL	0.619	0.493–0.735	174.5	45.0	87.5
	GFAP	0.614	0.451–0.698	163.81	64.0	70.0

AUC, area under the receiver operating characteristic curve; CI, confidence interval; Cutoff, optimal cut-off value (maximized Youden’s index); Sen, sensitivity; Spe, specificity; Aβ, amyloid-β; p-Tau: phosphorylated tau protein; NfL, neurofilament light chain; GFAP, glial fibrillary acidic protein.

In distinguishing AD from HC, plasma phosphorylated Tau showed excellent diagnostic performance. p-Tau217 performed best with an AUC of 0.894 (95% CI: 0.803–0.951); at the cut-off value of 2.17 pg./mL, the specificity was 100.0% and sensitivity was 70.0%. Notably, plasma p-Tau217 levels were significantly elevated in AD compared with both HC and CSVD (Bonferroni *post-hoc* test, both *p* < 0.001), indicating that peripheral p-Tau217 is a highly specific indicator for AD pathology. p-Tau181 ranked second with an AUC of 0.862 (95% CI: 0.764–0.930) and specificity of 96.7%, with significantly elevated levels in AD versus HC (*p* < 0.001). Although p-Tau217 and p-Tau181 demonstrated numerically higher AUC values than NfL (AUC = 0.832) and GFAP (AUC = 0.769), DeLong test revealed no statistically significant differences among these biomarkers (all *p* > 0.05), suggesting that the AD-specific phosphorylated Tau biomarkers, neurodegeneration marker NfL, and astrocyte activation marker GFAP all exhibited elevated expression in AD (all *p* < 0.001 versus HC) and contributed to the discrimination of AD from healthy controls.

In distinguishing CSVD from HC, the efficacy ranking of biomarkers changed significantly, reflecting a distinct pathological profile. NfL reflecting axonal injury showed the highest AUC of 0.886 (95% CI: 0.793–0.947), closely followed by GFAP reflecting astrocyte activation with an AUC of 0.881 (95% CI: 0.786–0.943). At the cut-off value of 36.39 pg./mL, GFAP achieved 95.0% sensitivity and 70.0% specificity. Consistent with the ROC findings, both NfL and GFAP levels were significantly elevated in CSVD compared with HC (Bonferroni post-hoc test, both *p* < 0.001). p-Tau181 also showed moderate diagnostic value (AUC = 0.762) with significantly higher levels in CSVD versus HC (*p* < 0.001). In contrast, AD core indicators p-Tau217 (AUC = 0.718) and Aβ1-40 (AUC = 0.526) showed limited diagnostic value for CSVD, which was further supported by the absence of significant differences in their plasma levels between CSVD and HC (both *p* > 0.05, Bonferroni *post-hoc* test). These findings indicate that chronic cerebral ischemia in CSVD primarily drives astrocyte-mediated neuroinflammation and axonal injury rather than AD-specific amyloid or Tau pathologies.

In clinical differentiation between AD and CSVD, AD-specific biomarkers dominated again. p-Tau217 yielded the highest differential efficacy with an AUC of 0.877 (95% CI: 0.781–0.941), consistent with its significantly higher plasma levels in AD compared with CSVD (Bonferroni post-hoc test, *p* < 0.001). At the cut-off value of 3.07 pg./mL, it could distinguish AD from CSVD with a specificity of 85.0% and sensitivity of 52.5%. p-Tau181 (AUC = 0.782) and Aβ1-40 (AUC = 0.733) also showed moderate differential ability; however, their plasma levels did not differ significantly between AD and CSVD (both *p* > 0.05), suggesting that their discriminatory power may be partially attributed to the contrast against HC rather than direct inter-disease differentiation. Notably, although NfL and GFAP performed well in distinguishing single disease from healthy controls, they showed poor efficacy in differentiating AD from CSVD (AUC = 0.619 and 0.614, respectively). This was consistent with the significant elevation of both NfL and GFAP in CSVD compared with HC (*p* < 0.001), which narrowed the expression gap between AD and CSVD for these neurodegeneration-related markers. Taken together, these results suggest that a single universal biomarker is insufficient for etiological differentiation between AD and CSVD, while plasma p-Tau217, characterized by its AD-specific elevation and minimal confounding by cerebrovascular pathology, has crucial application value in complex clinical differential diagnosis.

### 2.7 Correlation between cognitive score and plasma biomarkers

**Figure 3:**
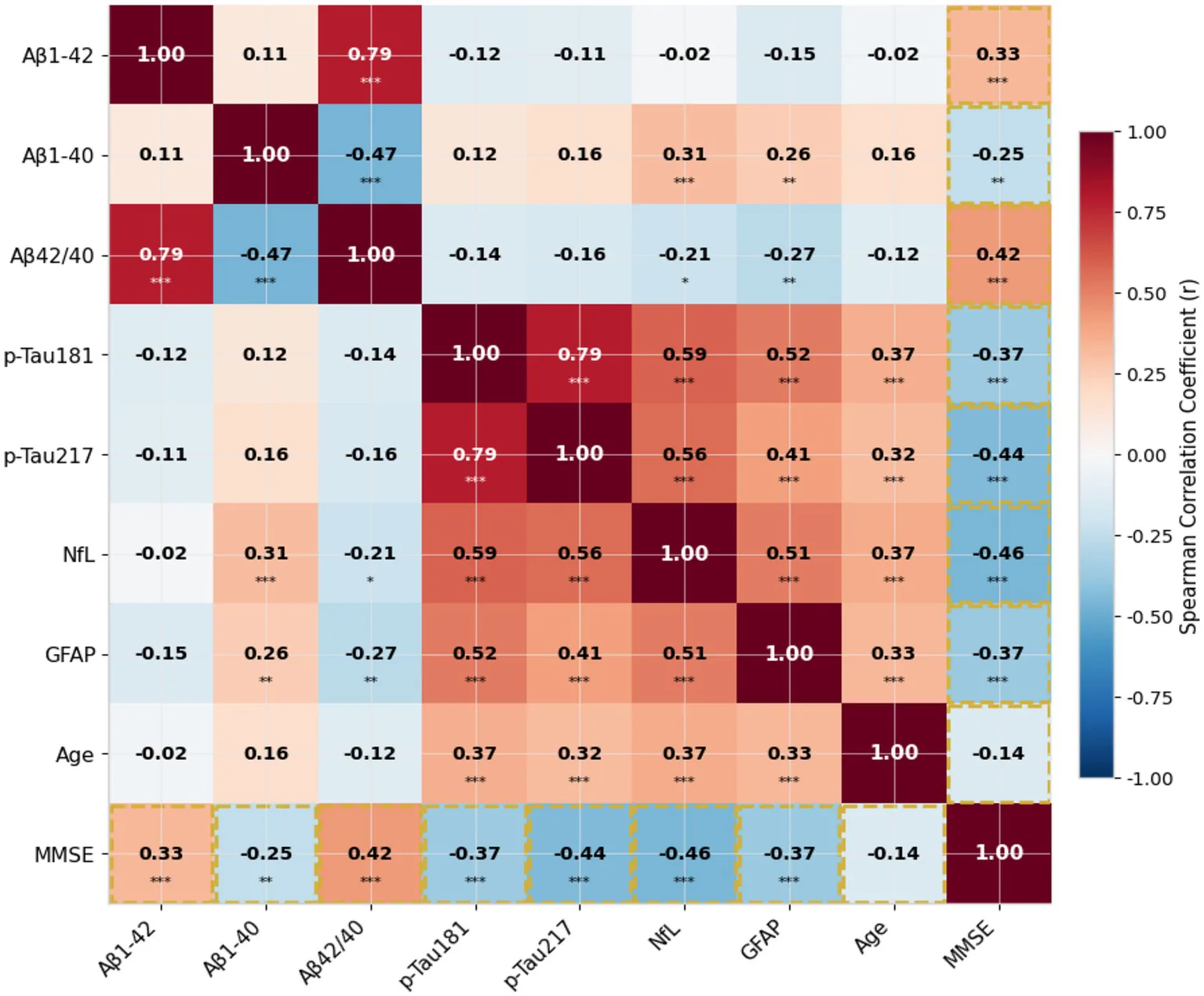
Spearman correlation heatmap of plasma biomarkers, age, and cognitive function (MMSE). This heatmap presents the Spearman correlation coefficients (*r*) among plasma biomarkers (A*β*1-42, Aβ1-40, Aβ42/40, p-Tau181, p-Tau217, NfL, GFAP), as well as their correlations with age and Mini-Mental State Examination (MMSE) scores. The color gradient from blue to red indicates correlation coefficients ranging from −1 (perfect negative correlation) to 1 (perfect positive correlation). Values within each cell represent the exact Spearman correlation coefficients. Regions framed with golden dashed lines highlight the correlations between all indicators and cognitive function (MMSE scores). *p < 0.05, **p < 0.01, ***p < 0.001. MMSE, Mini-Mental State Examination; Aβ, myloid-β protein; p-Tau, hosphorylated tau protein; NfL, eurofilament light chain; GFAP, lial fibrillary acidic protein.

As shown in the Spearman correlation heat map in Figure 3, we systematically analyzed the intrinsic correlations among plasma biomarkers, age, and global cognitive function assessed by the MMSE score.

The main findings were as follows. As highlighted by the gold dotted box in Figure 3, multiple peripheral biomarkers were significantly correlated with the degree of cognitive impairment. On the one hand, biomarkers reflecting axonal injury, Tau pathology and glial inflammation were significantly negatively correlated with MMSE scores. Among them, NfL showed the strongest negative correlation (*r* = −0.46, *p* < 0.001), followed by p-Tau217 (*r* = −0.44, *p* < 0.001). p-Tau181 (*r* = −0.37), GFAP (*r* = −0.37) and Aβ1-40 (*r* = −0.25) also exhibited significant negative correlations (*p* < 0.01 or *p* < 0.001). On the other hand, the Aβ42/40 ratio, which reflects amyloid pathology, showed the strongest positive correlation with MMSE scores (*r* = 0.42, *p* < 0.001), and Aβ1-42 was also significantly positively correlated (*r* = 0.33, *p* < 0.001), indicating that a higher peripheral Aβ proportion was associated with better cognitive performance.

The central area of the heat map revealed strong co-expression patterns among biomarkers of different pathological mechanisms. Firstly, a strong positive correlation was observed between p-Tau181 and p-Tau217 (*r* = 0.79, *p* < 0.001). Secondly, both p-Tau181 and p-Tau217 were significantly positively correlated with the neuronal injury marker NfL (*r* = 0.59, 0.56) and the inflammatory marker GFAP (*r* = 0.52, 0.41) (all *p* < 0.001). A close correlation was also found between NfL and GFAP (*r* = 0.51, *p* < 0.001). This clustered positive correlation network suggests a close cascade relationship among Tau pathology, neuronal degeneration and neuroinflammation during disease progression. In contrast, the Aβ42/40 ratio showed weak negative correlations with NfL and GFAP (*r* = −0.21 and −0.27, respectively).

This study further demonstrated that aging is an important contributing factor to the elevation of neurodegenerative biomarkers. Age was significantly positively correlated with NfL and p-Tau181 (both *r* = 0.37, *p* < 0.001), as well as GFAP (*r* = 0.33, *p* < 0.001) and p-Tau217 (*r* = 0.32, *p* < 0.001), indicating that the baseline levels of peripheral neuronal damage and glial inflammatory biomarkers gradually increase with advancing age.

## 3 Discussion

The present study systematically evaluated the expression differences and diagnostic efficacy of peripheral Aβ1-42, Aβ1-40, Aβ1-42/Aβ1-40, p-Tau181, p-Tau217, NfL and GFAP in AD and CSVD, and further analyzed their correlations with cognitive function. Distinct peripheral molecular signatures between AD and CSVD were clarified, providing critical evidence for early clinical identification, etiological classification and cognitive impairment assessment.

Firstly, peripheral blood of AD patients presented typical amyloid metabolic disorder and Tau hyperphosphorylation. Plasma Aβ1-42 decreased, Aβ1-40 slightly increased, and Aβ42/40 ratio declined specifically, which was consistent with previous studies (21, 22). Peripheral Aβ is susceptible to metabolic interference, while Aβ42/40 ratio can eliminate individual baseline differences and stably reflect central amyloid pathology (23–25). p-Tau217 showed excellent diagnostic performance for AD screening and differential diagnosis with CSVD, confirming that p-Tau217 is the most AD-specific peripheral biomarker free from cerebrovascular confounding factors (20, 26).

Secondly, the differential expression of GFAP and NfL revealed the specific neuroinflammatory and pan-neurodegenerative characteristics of CSVD, respectively. The present study yielded a finding of important clinical differential diagnostic value: plasma GFAP levels in the CSVD group were significantly higher than those in the AD and HC groups, with an AUC of up to 0.881 for distinguishing CSVD from HC, indicating that GFAP is an optimal biomarker for identifying CSVD. Previous studies have mainly focused on the role of GFAP in AD. By contrast, our findings confirmed that chronic cerebral ischemia can persistently activate astrocytes and induce intense neuroinflammation, resulting in specifically elevated peripheral GFAP expression in CSVD (27–31).

Under chronic cerebral ischemia and hypoperfusion, long-term cerebral hypoxia, ischemia and impaired energy metabolism trigger reactive astrocyte activation and upregulate GFAP expression. Concomitant oxidative stress, inflammatory responses, blood–brain barrier disruption and neurovascular unit injury facilitate the release of GFAP from brain tissue into the peripheral circulation, leading to elevated plasma GFAP levels (32, 33). In addition, chronic hypoperfusion may induce loss of AQP4 polarity and glymphatic system dysfunction, further promoting GFAP accumulation in the brain and its transport into the bloodstream. Therefore, plasma GFAP can serve as an important biomarker reflecting astrocytic injury and neuroinflammation associated with chronic cerebral ischemia (34, 35).

This finding effectively compensates for the clinical limitation that relying solely on Aβ and Tau cannot distinguish vascular cognitive impairment from neurodegenerative cognitive impairment. As a pan-neurodegenerative biomarker of axonal injury, NfL levels were highest in the AD group, followed by the CSVD group. Both neuronal apoptosis mediated by neurofibrillary tangles in AD and axonal degeneration induced by chronic ischemia in CSVD can lead to increased release of NfL (36). Although NfL lacks specificity for etiological differentiation, its significant negative correlation with MMSE scores establishes its role as a general indicator for evaluating the severity of neurodegeneration and cognitive decline (37).

Thirdly, biomarker correlation network reflected the pathological cascade of cognitive decline. The Aβ42/40 ratio was positively correlated with MMSE, while p-Tau, NfL and GFAP were negatively correlated with cognitive score. Strong correlations among these indicators revealed mutual promotion among Aβ deposition, Tau spread, inflammation and neuronal loss. Age-related elevation of these biomarkers suggested that age-corrected reference values should be adopted in clinical practice.

Finally, clinical phenotypic differences further supported the pathological classification value of these biomarkers. CSVD was characterized by hypertension, carotid atherosclerosis and severe white matter hyperintensities, while AD was featured by medial temporal lobe atrophy. Abnormal glucose and lipid metabolism were closely involved in cognitive impairment pathogenesis.

This study has several limitations: single-center cross-sectional design, limited sample size, lack of longitudinal follow-up, no CSF or PET gold standard comparison, and unadjusted lifestyle confounding factors. In addition, patients with moderate to severe CSVD burden were excluded from the AD group, while patients presenting typical clinical phenotype of AD were excluded from the CSVD group. This strict exclusion criteria created artificially pure disease subgroups. Considering that AD and CSVD frequently coexist among elderly patients with cognitive impairment, the diagnostic performance of plasma biomarkers observed in the present study may be overestimated, and these findings cannot be directly generalized to patients with AD-CSVD co-pathology. Further multicenter longitudinal studies are needed to promote clinical transformation of these plasma biomarkers.

## 4 Conclusion

This Chinese population-based study confirmed that among patients with isolated AD or isolated CSVD, plasma p-Tau217 is the optimal specific biomarker for AD diagnosis and AD-CSVD differentiation. GFAP is a key marker for identifying CSVD and distinguishing vascular from degenerative cognitive impairment. NfL can quantify the overall severity of pan-neurodegeneration, and Aβ42/40 ratio serves as an auxiliary indicator for reflecting AD amyloid pathology. Caution is needed when extending these conclusions to patients with AD-CSVD co-pathology. Combined detection of multi-biomarker panel can realize non-invasive early screening and precise etiological classification for cognitive impairment in individuals without mixed AD and CSVD pathologies.

## Data Availability

The original contributions presented in the study are included in the article/supplementary material, further inquiries can be directed to the corresponding author.
